# Ergonomic Design and Evaluation of Cloth-Pulling Devices for Praewa Silk Weavers 

**DOI:** 10.12688/f1000research.163622.2

**Published:** 2025-06-16

**Authors:** Wuttichai Yota, Manida Swangnetr Neubert, Teeraphun Kaewdok

**Affiliations:** 1 Department of Medical Engineering, Thammasat School of Engineering, Thammasat University, Khlong Luang, Pathum Thani, 12121, Thailand; 2 Human-Robot Collaboration and Systems Integration Research Unit, Chulalongkorn University, Bangkok, Bangkok, 10330, Thailand; 3 Department of Industrial Engineering, Faculty of Engineering,, Chulalongkorn University, Bangkok, Bangkok, 10330, Thailand; 4 Thammasat University Research Unit in Occupational Ergonomics, Thammasat University, Khlong Luang, Pathum Thani, 12121, Thailand; 5 Faculty of Public Health, Thammasat University, Khlong Luang, Pathum Thani, 12121, Thailand

**Keywords:** Informal workers, Well-being, Safety, Decent work, Hand tools, Muscular effort, Electromyography

## Abstract

**Background:**

Traditional weaving professionals pull cloth manually during the handloom process, which can lead to several unnoticed musculoskeletal disorders. The aim of this study was to design and evaluate the effectiveness of cloth-pulling devices for Praewa silk weavers in Thailand.

**Methods:**

An experimental trial was conducted using surface electromyography to evaluate weavers’ muscle activity, productivity and perceived satisfaction during the Praewa silk-pulling process while employing traditional cloth pulling, using a standard cloth-pulling device and using an ergonomic prototype cloth-pulling device.

**Results:**

The results showed that the levels of muscle activity and hand activity with the prototype design were generally lower than those with traditional cloth pulling and with the standard cloth-pulling device (p < 0.01). There was a significant preference for the prototype, based on productivity and perceived satisfaction (p < 0.01).

**Conclusions:**

The new cloth-pulling device was found to be applicable and acceptable among the weavers. Further research is needed to make appropriate revisions to the ergonomically designed tool based on ergonomic measurements of muscle activity and fatigue under field production conditions. Studies related to workstations and working conditions are needed in the weaving industry sector.

## Introduction

The informal labour sector is considered the largest labour force in Thailand. According to a 2023 labour force survey by the National Statistical Office, there were 18.47 million (31.35%) people outside the labour force in Thailand, of whom skilled workers making up 10.68% of this group. The safety of this sector’s working environment has decreased, with rates of 7.21 % and 6.64 % recorded in 2022 and 2023, respectively. ^1^ The profession of producing Prawa silk, which is considered a unique craft of the Phu Thai people, is renowned and recognised by both governments and the private sector at the national and international levels. It is a traditional occupation of villagers in northeastern Thailand, with the majority located in Kalasin Province. The artisans who produce Praewa silk create pieces that are typically 1 wah (2 cm) or one arm’s length in size. Each piece takes between 3 months and 1 year to make. On average, artisans weave for approximately 3–6 hours per day and produce approximately 3–5 cm of silk, depending on the complexity of the pattern. ^2^ This leads to an increased risk of work-related musculoskeletal disorders (WMSDs), particularly ergonomic problems, and there are significant cases of musculoskeletal disorders (MSDs) among professionals in this occupation. According to a previous survey working condition among weavers, working more than 8 hours a day was found to account for 97.40% of MSD cases. Regarding work-related stress, 54.80% of weavers experienced occasional stress, 20.00% experienced frequent stress and 16.10% reported no work-related stress. ^3^ A quarter of the weavers report that their pain has caused them to stop working for 1–30 days. Age, years of experience and daily hours worked all have an impact on the prevalence of WMSDs, as do extended workdays, awkward postures and repetitive limb movements. ^4^ The prevalence of MSDs among handloom weavers in Thailand who use the traditional handloom weaving technique has been found to be the highest, at shoulder (91.43%), lower back (85.71%) and wrists (60.00%), respectively. ^5^ In a study by Daneshmandi et al. ^6^ found that the prevalence of WMSDs and ergonomic risk factors among assembly line workers. The results for MSDs were mostly associated with the lower back (73.6%), wrists/hands (71.7%) and neck (67.9%). Most (80%) of the working postures analysed using the Rapid Upper Limb Assessment (RULA) were at action levels 3 or 4. ^6^ Kaewdok ^7^ stated that ergonomic risk factors, such as work posture, behaviour, tools, equipment and the working environment, can lead to injuries when there is a mismatch between the workload and the worker’s abilities and limitations. This is particularly true for WMSDs. In Thailand, these disorders have resulted in up to 3.8 million lost workdays due to medical treatment in 2020. ^1^ According to occupational health reports, the most common occupational illness, accounting for 45% of cases of MSDs. The most common problem encountered by informal sector workers in the work occupational was repetitive motion at work for 39.9% of workers. ^1^ A study of Besharati found that the highest rate of prevalence of MSDs within the last 12 months. ^8^ In the case of Praewa silk weaving, the work involves repetitive hand movements to pull silk threads into patterns, requiring weavers to spend prolonged periods pulling individual threads while maintaining the same posture. This process includes wrist rotations during the insertion of threads to create patterns in the fabric. Furthermore, no supportive tools are available to alleviate physical strain during the weaving process. Therefore, it is essential to design an ergonomic handle. ^9^

Ergonomics is an interdisciplinary field that studies human characteristics, capabilities and limitations to optimise work conditions. It includes job characteristics, posture, tools and equipment to ensure that workers are comfortable, safe and have a good quality of life with minimal injuries and accidents. Ergonomics also involves studying the relationship between workers and their work environments. ^10^ This concept is combined with user-centred design principles through the design thinking process, which consists of five steps: understanding behaviour, defining the problem, ideating, prototyping and finally testing and evaluating. ^11^ This aligns with the capabilities of workers in Thailand as the country advances towards Industry 4.0. This era marks a transformation in industrial systems through the adoption of advanced technology and automation to produce goods more quickly, efficiently and with higher quality. Key factors for evaluating worker capabilities include adherence to the 17 Sustainable Development Goals (SDGs) aimed at promoting sustainable development globally. The goal for worldwide good health promotes healthy lifestyles, preventive measures and modern, efficient healthcare for everyone aligns with SDG 3 – to ensure healthy lives and promote well-being for all at all ages by fostering good health and well-being across all age groups – and also supports SDGs 8: to promote sustained, inclusive economic growth through improved labour productivity and the development of technology and innovation in production. This goal is full and productive employment with meaningful work for everyone by 2030 with established by the United Nations in 2015. ^12^ The production of Praewa silk by the Phu Tai community in Ban Phon has experienced significant growth in both quantity and quality. This traditional textile is distinguished by its intricate patterns and substantial dimensions. The price of Praewa silk typically ranges from 15,000 to 40,000 baht per piece. However, certain designs that require a high level of craftsmanship and take approximately 5 to 6 months to complete can command prices between 50,000 and 100,000 baht.

It is essential to design cloth-pulling devices for Praewa silk weavers that reduce the risk of wrist injuries, such as carpal tunnel syndrome, De Quervain’s tendonitis and trigger finger. Proper ergonomic design for silk-pulling tools can help align with workers’ physiology, enhance safety and improve overall work efficiency. ^13^ By adhering to ergonomic principles, these tools can reduce strain and potential injuries, leading to safer and more effective working environments. ^14^ The novelty of this research lies in the design of a Praewa silk cloth-pulling devices that adheres to ergonomic principles, specifically tailored for Praewa silk weavers. This innovation aims to enhance worker safety and improve operational efficiency by aligning with the physical capabilities of the users. Furthermore, the device is expected to reduce work-related musculoskeletal discomfort and increase overall production capacity. The design of cloth-pulling devices based on ergonomics can help alleviate worker discomfort and increase productivity. This study aims to design and evaluate ergonomic cloth-pulling devices for Praewa silk weavers to enhance production efficiency and reduce the risk of MSDs through proper ergonomic principles. The study aims to design an ergonomics-based cloth-pulling device and to evaluate the effectiveness of such devices for Praewa silk weavers.

## Methods

### Participants

Twenty-nine female Praewa silk weavers volunteered to participate in this study. The sample size was calculated using the mean maximum voluntary contraction (MVC) among handloom weavers from a previous study. ^5^ The participants were aged between 40 and 70 years (mean = 55.28; SD = 7.99); all were right-handed and none had a history of bone dislocation or injury to the upper-body extremities. Before taking part in the experimental procedure, each participant was briefed about the purpose of the study. Informed consent was obtained from all participants.

### Hand anthropometric measurements

**Figure 1.**
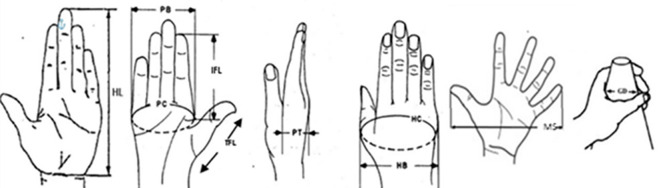
Hand anthropometric dimensions.

In this study, 10 hand anthropometric dimensions were measured for each participant. These dimensions were chosen based on their usefulness relative to the design considerations for cloth-pulling devices, with an emphasis on comfort and safety. Based on the hand proportion measurements, the cloth-pulling devices were designed as lightweight plastic hooks shaped like a parrot’s beak. The dimensions of the tools were determined from 10 hand measurements: hand length, palm breadth, palm circumference, thumb finger length, index finger length, palm thickness, hand circumference, hand breadth, maximum spread and grip diameter ( Figure 1).

Details of the hand anthropometric measurement are shown in Table 1.

**Table 1.** The 10 hand anthropometric measurements of the study participants (n = 29).

Hand anthropometric dimensions *	Percentiles			Mean (min–max)	SD	CV
	5 ^th^	50 ^th^	95 ^th^			
Hand length	15.50	14.70	16.20	14.78 (11.50–17.30)	0.82	5.55
Palm breadth	4.60	5.50	6.40	5.51 (4.00–7.20)	0.56	9.98
Palm circumference	15.20	17.80	21.10	17.79 (14.10–21.40)	1.83	10.29
Thumb finger length	5.60	6.20	7.10	6.31 (5.20–7.90)	0.49	7.77
Index finger length	4.70	5.20	7.00	5.43 (4.10–8.70)	0.63	11.60
Palm thickness	1.40	1.60	1.80	1.60 (1.30–1.90)	0.133	8.13
Hand circumference	16.40	20.00	23.10	19.73 (15.60–23.80)	2.15	10.90
Hand breadth	5.60	6.50	8.21	6.66 (4.20–9.00)	0.75	7.83
Maximum spread	13.70	14.75	17.50	15.21 (12.80–24.00)	1.46	10.36
Grip diameter	3.20	4.10	4.60	4.05 (3.00–5.20)	0.36	8.64

* All dimensions are in cm.

### Prototype cloth-pulling device design

**Figure 2.**
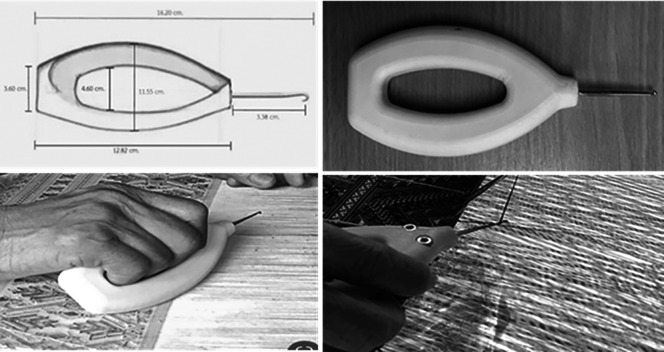
Ergonomic prototype cloth-pulling device.

Based on the results of the walkthrough survey phase, pulling cloth manually, as the most commonly used approach in Praewa silk weaving operations, was of central attention. The following design procedure was followed:

• Understanding behaviour: Interactions were examined among the weavers’ characteristics, work conditions, tools/equipment and health effects. A collection of the hand tools currently used for Praewa silk weaving was provided.
• Defining the problem: The problem was clearly defined based on user feedback and ergonomic analysis. A problem statement was formulated to address the specific ergonomic issues identified.
• Ideating: Hand tools for Praewa silk weaving were studied during operation to consider the design/redesign requirements and determine the specifications, including weight, texture, handle shape and hand posture during application.
• Prototyping: New models of cloth-pulling devices were developed according to the hand anthropometrics of the weavers and ergonomic design principles to reduce the risk of MSDs during the weaving task. The cloth-pulling devices’ handles were made of plastic polyester materials. Handle length was calculated using the 95th percentile of hand length (16.20 cm), handle width using hand circumference (23.10 cm) and handle diameter using grip diameter (4.60 cm) ( Figure 2).
• Testing and evaluating: A usability test was conducted to assess the comfort and applicability of the hand tools designed for cloth-pulling and the weavers’ perceptions of them.

### Experimental design

All the participants were randomly divided into three cloth-pulling groups for the experimental trial: (A) traditional cloth pulling, (B) using a standard cloth-pulling device and (C) using an ergonomic prototype cloth-pulling device. Each technique was carried out for 40 minutes. Before starting the experimental trial, the participants were trained to pull cloth using the newly designed devices to avoid errors. The instrument used and the measurement of the variables in this study are described below:

• Surface electromyography (EMG): EMG signals were recorded during the trials using an eego ^TM^ amplifier (EE – 223, revision no. 1.2, Germany). Four channels were used to simultaneously record the EMG activity of four muscles – the upper trapezius, the biceps brachii, the brachioradialis and the flexor carpi ulnaris. ^15, 16^ These muscles were selected based on their relevance to the weaving task performed, in accordance with a previous study. ^17, 18^ Skin was prepared using a cotton alcohol swab before surface electrodes were placed on the muscles. The electrodes were placed on the muscles according to the reviewed literature. ^19, 20^ Bipolar surface electrode placement (disposable Ag/AgCl) with a 2-cm interelectrode distance was performed in accordance with recommendations found in the literature. ^21– 23^
• MVC: Muscle activity was normalised using MVC, which was measured for each muscle at the beginning of the experiment. The root mean square (RMS) values of the surface EMG data (millivolts) of all the muscles were determined for muscle strength and fatigue. Three MVC efforts were performed for each muscle for 5 seconds with three repetitions, with 20 minutes of rest provided between the trials to eliminate traces of muscle fatigue. In all of the tests performed, pressure and resistance were applied by the participants to the respective muscle tendons. ^18^ Average RMS values were considered for subsequent normalisation of the muscles (upper trapezius, biceps brachii, brachioradialis and flexor carpi ulnaris).
• Ergonomic observational risk assessment: HAL and normalised peak force were used to identify the level of hand activity on a scale of 0 to 10, where zero is virtually no activity and 10 is the highest imaginable hand activity. ^24^ Weavers’ HALs were calculated based on observations and interviews regarding their daily exposure duration while weaving Prawa silk. Then, the threshold limit values were grouped into three classifications (below the action value of 0.56, between 0.56 and 0.78 and above the threshold limit value of 0.78). ^25– 27^
• Productivity test: Upon completion of each trial, the Prawa silk length was measured using measurement tape (in cm) to evaluate productivity from the different cloth-pulling hand-tool devices.
• Perceived usability: To evaluate the user’s experiences when interacting with the cloth-pulling devices, after they had finished weaving, the participants were asked to self-assess their perceived satisfaction between the different models. Each item had a response set using a five-point Likert scale ranging from 1 to 5 (1 = strongly disagree, 5 = strongly agree). ^28, 29^

**Figure 3.**
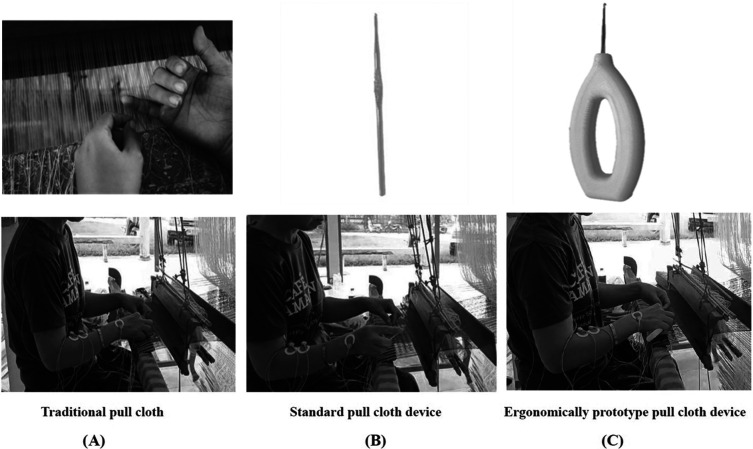
(A) Traditional cloth pulling. (B) Using a standard cloth-pulling device. (C) Using an ergonomic prototype cloth-pulling device.

In this study, three different cloth pulling devices were used (traditional cloth pulling, a standard cloth-pulling device and an ergonomic cloth-pulling device) ( Figure 3).

### Statistical analysis

The data analysis was carried out using SPSS Statistics software (v. 23). Descriptive statistics, including number, percentage, percentile, mean and standard deviation (SD), were used to describe the participants’ characteristics, MVC, HAL level, productivity and perceived usability. A normality test was performed to determine whether the value sets were normally distributed, for which a Shapiro–Wilk test was carried out to certify its validity. A one-way ANOVA test was then performed to determine the effects of the cloth-pulling devices (A vs. B vs. C) on the dependent variables. Pairwise comparison tests were conducted using a post hoc test with Tukey’s adjustment. A p-value below 0.05 was considered statistically significant.

## Results

### Muscle activity

**Figure 4.**
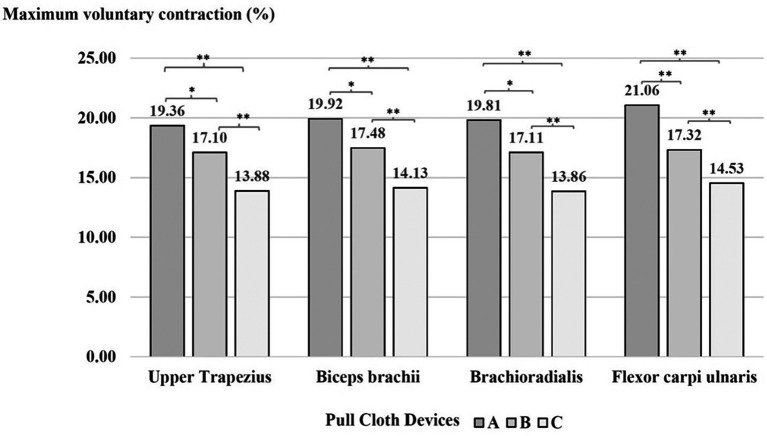
Effect of the cloth-pulling devices on muscle activity (normalised %MVC) while performing the simulated Praewa silk task. *p < 0.05, **p < 0.01.

According to the ANOVA results, the main effects of the cloth-pulling devices were statistically significant for muscle activity. The result of Tukey’s post hoc test indicated that weaving with device A required significantly higher exertion levels (%MVC) in the upper trapezius, biceps brachii and brachioradialis compared with device B (p < 0.01) and the flexor carpi ulnaris (p < 0.05). Higher muscle activity was recorded for device B than for device C (p < 0.01) and for device A than for device C (p < 0.01) in each muscle, as shown in Figure 4.

### Hand activity level

**Figure 5.**
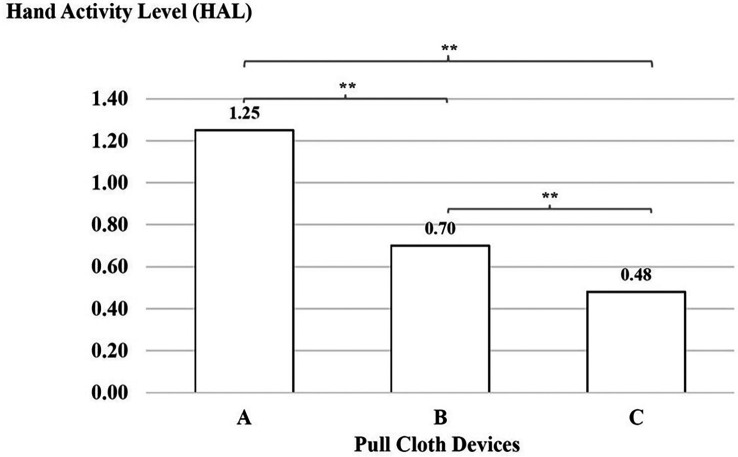
Hand activity level using the three cloth-pulling devices. **p < 0.01.

There was a significant difference with regard to the force ratings, which were higher for device A than for device B, for device B than for device C and for device A than for device C (p < 0.01), as shown in Figure 5.

### Productivity test

**Figure 6.**
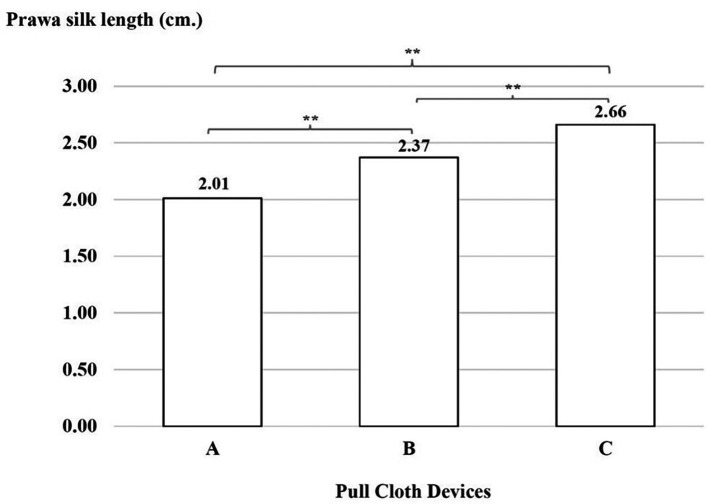
Prawa silk length (in cm) using three cloth-pulling devices. **p < 0.01.

There was a significant difference with regard to Prawa silk lengths, which were shorter with device A than with device B, with device B than with device C and with device A than with device C (p < 0.01), as shown in Figure 6.

### Perceived usability

**Figure 7.**
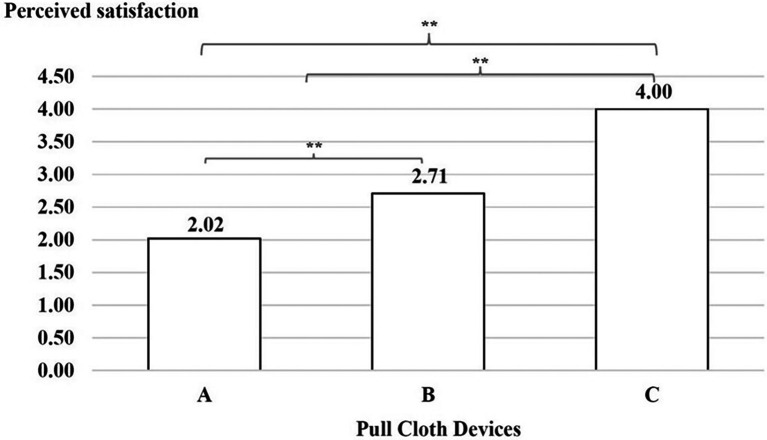
Perceived satisfaction with the three cloth-pulling devices. **p < 0.01.

A comparison of the mean of perceived satisfaction between devices A, B and C was conducted. The results indicated that the users’ experiences were lower when interacting with device A than with device B, with device B than with device C and with device A than with device C, as shown in Figure 7.

## Discussion

Hand tools are widely used in a large number of occupations, with many workers having to use them to accomplish their duties. ^30^ This study dealt with the effect of three different cloth-pulling devices in weaving Praewa silk (traditional cloth pulling, a standard cloth-pulling device and an ergonomic prototype cloth-pulling device). In this study, a cloth pulling design with a new handle was considered an important factor in the safe, comfortable and easy use of hand tools among Praewa silk weavers. ^31^ The results showed that the cloth-pulling devices had a significant effect on the activity of each muscle. When performing the Praewa silk task with the prototype cloth-pulling device (device C), the muscular activity (% MVC) of each muscle was generally lower than with traditional cloth pulling (device A) and the standard cloth-pulling device (device B). This is in agreement with the reduction in muscle activity observed in Shankar et al., ^32^ which found that ergonomically optimised hand tools can decrease muscle activation patterns by 15–25% compared to traditional designs. Previous studies have confirmed a lower muscle activity level when working with the new hand-tool design. ^21, 29, 32^ It is possible that muscle fatigue reduced the muscles’ ability to generate force due to exhaustion and strenuous activity. Over-contraction of the muscles leads to the development of fatigue due to an inadequate blood supply to the muscles that inhibits the supply of oxygen, resulting in increased production of lactic acid in the blood and further affecting work performance, with a reduction in work capacity and productivity. ^33^ The results of the evaluation trials showed that the muscle activities of the biceps brachii and flexor carpi ulnaris showed higher maximum contraction (%MVC) compared to the other muscles. This may be attributed to the nature of the task of cloth pulling, which generally requires repetitive pulling actions, with the flexor muscles of the forearm used during the weaving process. In addition, the two muscle groups were demonstrated to be the most active among the arm muscles because of their upper-extremity positions during lifting, handling, pushing and pulling tasks. ^34^ Due to the dominance of the pulling action and other factors, such as elevated arm posture and repetitive movement of the hand during the manual weaving task, this study’s finding that the ergonomic prototype cloth-pulling device could minimise the risk of muscle exertion during the performance of weaving tasks seems appropriate. The performance of the worker’s muscle strength and proper hand tools was considered.

Weaving involves a variety of tasks that are performed while the weaver sits constantly in a static position while making repeated movements of the upper limbs to operate shuttles, with the arms extended away from the body. ^14, 35^ The results of this study indicate that the decreased HAL with the new cloth-pulling device reduces hand activity and the level of effort required for a typical posture while performing a cloth-pulling task. During work with the cloth-pulling prototype, the HAL had a lower action limit value with indicated acceptable tasks. These findings support previous studies showing that optimised tool designs can significantly reduce repetitive hand movements and associated strain. ^26, 36, 37^ This may highlight that a lower HAL indicates a reduced risk of developing work-related musculoskeletal disorders, especially in the hands and wrists, during extended weaving operations. Prolonged static postures in traditional looms have been linked to high rates of musculoskeletal issues among handloom weavers. ^38^ Ergonomic tools help maintain optimal HAL, potentially increasing productivity and reducing injury risk. The findings emphasise the importance of improved work environments and postures for enhanced safety and lower injury risk. Erdem and Savaş’s study ^39^ identified significant risk factors for WMSDs, including chronic disease, hand tool usage and high RULA scores, as well as unawareness of ergonomic risks among those with no prior accidents. A previous study by Rahman et al. showed a decrease in RULA scores and a reduction in upper-limb disorder risk from high to low within 6 months of using the new loom design, with high-risk weavers dropping from 12.5% to 2.5%. ^40^ This evidence underscores the effectiveness of ergonomic improvements in reducing MSD risks among weavers.

This study focused on designing a cloth-pulling device for a common procedure in Praewa silk weaving. The implementation of the ergonomic cloth-pulling device led to notable improvements in user comfort and productivity. The results of the usability test indicated that there was a significant difference with regard to length, with the silk produced using the ergonomic prototype cloth-pulling device being longer than that produced using the standard device as well as traditional cloth pulling. Moreover, the results also showed that the mean perceived satisfaction with the ergonomic prototype cloth-pulling device was the highest. This may indicate that the users’ experiences when interacting with the new hand-tool device were perceived as satisfying. It can be concluded that the ergonomic prototype cloth-pulling device is suitable in terms of comfort and applicability for weavers. This is consistent with a previous study, ^41^ which concluded that an ergonomic design can significantly improve workplace health and productivity. User satisfaction was strongly related to reduced physical strain and improved work efficiency. This may be because the positive feedback from weavers regarding comfort and ease of use suggests that the ergonomic improvements effectively addressed the weavers’ practical needs and preferences. ^30, 31^ This is in agreement with the results of other studies showing that ergonomically well-designed hand tools may reduce the risk of occupational injuries to the upper limbs. ^21, 32^ Such tools also provide comfortable work for users and provide high product quality to consumers. ^11, 42^

Hand tools are the primary instruments in a large number of industrial tasks, and most workers use them to accomplish activities that must be done through manual operation. ^11, 23, 32, 42^ The results revealed that due to the proposed ergonomic design principle, the ergonomically designed device reduced the mean values of muscle activity during each of the proposed tasks. This is particularly important considering the high prevalence of injuries and MSDs among workers involved in hand-tool use. Placing emphasis on reducing muscular effort is therefore an essential step in avoiding such problems. This study emphasises the importance of adjusting grip diameters and considering the ergonomics of silk weaving workstations, which not only helps prevent health issues but also boosts productivity among weavers. The ergonomic cloth-pulling device in this study was shown to enhance productivity, reduce MSDs and promote better posture. Its key ergonomic principles include maintaining a neutral wrist position, minimising the grip force requirements, avoiding pressure points on the palmar surface and distributing forces evenly across the hand through the handle design. ^11, 41, 43^ However, the application of ergonomic principles to traditional weaving tools must consider both the physical requirements of the task and the anthropometric characteristics of the local weaving population. Therefore, further studies applying ergonomic principles and using worker-specific anthropometric data are needed to improve workstation design to enhance safety, comfort and overall work performance in the weaving industry.

A limitation of this study is that it focused only on Praewa silk weavers in Kalasin province in northeastern Thailand who used hand tools. There may be limitations in applying the results of this study to populations in other areas due to differences in the society, culture and body size proportions in each region. This is because fabrics, which come in various sizes and forms, depend on the needs and purposes for which they are used. The experience of weaving Praewa silk varies from individual to individual. Familiarity with different weaving patterns leads to different sitting postures when using each weaving tool. The experience of using each type of equipment also varies.

## Conclusion

This study showed that upper limb muscle activities occurred at a high rate among Praewa silk weavers. The experimental results showed improvements in terms of comfort and reduced muscle activity with the ergonomic prototype cloth-pulling device. Its design led to a significant reduction in the levels of hand activity and muscle activity for each muscle. Based on the usability test, it can be concluded that the new pull device was perceived as more comfortable and able to weave longer pieces than the other devices. The new ergonomically designed device was found to be applicable to and acceptable among the Praewa silk weavers.

However, further studies are required to make appropriate revisions to the ergonomically designed tool based on quantitative measures of musculoskeletal loading. Further studies related to workstations and work conditions are needed to consider weaving industry sectors.

## Ethical considerations

The study was approved by the Human Research Ethics Committee at Thammasat University (Science) (code 66EN108; COA No. 100/2566). The date of ethical approval received is October, 27, 2024.

## Consent for participate

A written informed consent was taken from all participants for their voluntary participation in the study prior to data collection explaining the possible use of the data for research and publication without revealing their identity.

## Data Availability

Figshare: “Ergonomic Design and Evaluation of Cloth-Pulling Devices for Praewa Silk Weavers” https://doi.org/10.6084/m9.figshare.28874267.v2 ^44^ This project contains the following underlying data:
- Law data Result and Assessment - A questionnaire is a set of questions used to gather information from individuals, typically for research. Data are available under the terms of the Creative Commons Attribution 4.0 International license (CC-BY 4.0). Ergonomic Design and Evaluation of Cloth-Pulling Devices for Praewa Silk Weavers © 2025 by Teeraphun Kaewdok is licensed under CC BY 4.0.
