# Supplementary material for: Ergonomic Design and Evaluation of Cloth-Pulling Devices for Praewa Silk Weavers 
Source: F1000Res. 2025 Sep 5;14:477. Originally published 2025 May 6. [Version 3] doi: 10.12688/f1000research.163622.3 (PMC12449686; doi:10.12688/f1000research.163622.3)
Supplement: Peer review history

# Reviewer report: Reviewer response for version 3

**DOI:** 10.5256/f1000research.187385.r412614

**Published:** 2025-09-24

**Authors:** Wisit Thongkum

**Affiliations:** 1 Mahasarakham University, Maha Sarakham, Thailand

After reading the article, it was found that the author's explanation was still unclear, especially in the methodology section. Therefore, the author should provide additional clarification for greater accuracy and clarity.

1. From introduction part: Praewa silk create pieces that are typically 1 wah (2cm) oronearm’s length in size. The conversion you've provided is incorrect.
2. The objectives of the study should be written clearly, or they can be combined into a single sentence.
3. When using purposive sampling in a research methodology section, it's crucial to be transparent about the selection process. How it was done: You need to specify the criteria used to select participants. Purposive sampling is a non-probability method where you deliberately choose individuals because they have specific characteristics relevant to your research question. You should detail inclusion and exclusion criteria.
4. Postural risk assessment:  average grand total score of 6.89±0.31, suggesting a relatively high level of risk. This part need reference or standard.
5. Prototype cloth-pulling device design using the Design Thinking method. It should be clearly specified in which stages the 29 participants were involved.
6. Several of your references do not adhere to the required citation format, as they lack page numbers. You may find using a citation management tool like EndNote helpful for correcting these.

Is the work clearly and accurately presented and does it cite the current literature?

Yes

If applicable, is the statistical analysis and its interpretation appropriate?

Yes

Are all the source data underlying the results available to ensure full reproducibility?

Yes

Is the study design appropriate and is the work technically sound?

Partly

Are the conclusions drawn adequately supported by the results?

Yes

Are sufficient details of methods and analysis provided to allow replication by others?

Yes

Reviewer Expertise:

Occupational health & safety and Environmental Technology

I confirm that I have read this submission and believe that I have an appropriate level of expertise to confirm that it is of an acceptable scientific standard.

# Reviewer report: Reviewer response for version 3

**DOI:** 10.5256/f1000research.187385.r411888

**Published:** 2025-09-19

**Authors:** Ashish Kumar Meher

**Affiliations:** 1 Central University of Jharkhand, Ranchi, Jharkhand, India

The comments and suggestions raised earlier in this manuscript have been incorporated, and hence, the manuscript may be accepted for indexed now in its present form. However, language editing is still required, as some of the sentences are unclear and incomplete.

Is the work clearly and accurately presented and does it cite the current literature?

Yes

If applicable, is the statistical analysis and its interpretation appropriate?

Yes

Are all the source data underlying the results available to ensure full reproducibility?

Yes

Is the study design appropriate and is the work technically sound?

Yes

Are the conclusions drawn adequately supported by the results?

Yes

Are sufficient details of methods and analysis provided to allow replication by others?

Partly

Reviewer Expertise:

Economics, Development economics, informal sector, handloom industry, occupational health issues

I confirm that I have read this submission and believe that I have an appropriate level of expertise to confirm that it is of an acceptable scientific standard.

# Reviewer report: Reviewer response for version 2

**DOI:** 10.5256/f1000research.183554.r395096

**Published:** 2025-08-22

**Authors:** Shanel Wu

**Affiliations:** 1 School of Information Technology, Carleton University, Ottawa, Ontario, Canada

Summary: This study was conducted with handweavers in northeastern Thailand who work in a traditional regional style (Praewa silk). Weavers often work long hours using repetitive motions and awkward positions, which leads to injury, pain, and chronic musculoskeletal disorders. The authors recruited a group of 29 female weavers to study how to improve their workplace's safety conditions by redesigning the cloth-pulling device (often a conventional crochet hook), which is a key hand tool in the process.

 Context: As previous reviewers have already commented on the quantitative results' presentation (which I note that I don't see many differences from version 1), I will focus more on how the paper contextualizes the problem and reports on the design process. I am proficient in handweaving with a variety of techniques, so I feel that I can more effectively comment on these aspects.

 Study Design (Q2): Overall, I appreciate that the researchers worked directly with weavers from this community and familiarized themselves with their craft. Suggestion(s): 

 > Because I'm also a weaver, I can somewhat able visualize how the pull cloth device is used, but many readers will need more clarification. I would add onto Figure 3 (or create a new figure) with more photos similar to 3A (top) of how their hands/tools are interacting with the yarns on the loom. Arrows to indicate direction of movements would help even more.

 > The first stage of the design process (Understanding behaviour) mentions collecting the hand tools that weavers currently use. I would be interested in seeing a picture of other tools besides the cloth pull devices, like the details of their handlooms, their sitting/standing postures while working, etc.

 Methods (Q3): ****Sharing their dataset and questionnaires will be extremely valuable for other researchers to translate the design improvements to other weaving communities and regions. Suggestion(s): 

 > The design process should be documented in more detail, especially the Prototyping stage. How many iterations did the prototype undergo? What changes were made between iterations and how did the researchers decide on what to change?

 > In contrast, the Evaluation aspects of the study are well-detailed, but think the timeline could be clearer since design work often includes some formative evaluation (e.g. between iterations). Did the authors test any of the earlier versions with weavers during prototyping (even informally)? 

 Source Data (Q5): ****As I noted previously, I really appreciate the data and documentation already included. My suggestions focus on what additional information is needed to replicate the final tool design. Suggestion(s):

 > Include any CAD files that were used in prototype fabrication and list software tools.

 > Bill of materials for the prototype with sources of components/materials.

 Other Suggestions: There are some revisions from previous reviews that have not been addressed.

 On the whole, very interesting research! I agree with the authors that handcraft ergonomics are under-researched. I look forward to seeing more work in this area, as there are many opportunities for further research, design improvements, and new community collaborations.

Is the work clearly and accurately presented and does it cite the current literature?

Yes

If applicable, is the statistical analysis and its interpretation appropriate?

Yes

Are all the source data underlying the results available to ensure full reproducibility?

Partly

Is the study design appropriate and is the work technically sound?

Yes

Are the conclusions drawn adequately supported by the results?

Yes

Are sufficient details of methods and analysis provided to allow replication by others?

Partly

Reviewer Expertise:

Human-computer interaction, human-centred design, textile crafts, digital fabrication, creativity support tools, craft and technology

I confirm that I have read this submission and believe that I have an appropriate level of expertise to confirm that it is of an acceptable scientific standard, however I have significant reservations, as outlined above.

# Response

**Published:** 2025-08-28

**Authors:** Teeraphun Kaewdok

**Affiliations:** Faculty of Public Health, Thammasat University, Khlong Luang, Pathum Thani, Thailand

Dear Reviewer, 

 Thank you very much for taking the time to review this manuscript. Please find enclosed our revised manuscript. We thank you the reviewers for their comments. We have revised the manuscript accordingly and provide specific answers.

 Reviewers' comments:

 Study Design (Q2): Overall, I appreciate that the researchers worked directly with weavers from this community and familiarized themselves with their craft. Suggestion(s):

 > Because I'm also a weaver, I can somewhat able visualize how the pull cloth device is used, but many readers will need more clarification. I would add onto Figure 3 (or create a new figure) with more photos similar to 3A (top) of how their hands/tools are interacting with the yarns on the loom. Arrows to indicate direction of movements would help even more.

 > The first stage of the design process (Understanding behaviour) mentions collecting the hand tools that weavers currently use. I would be interested in seeing a picture of other tools besides the cloth pull devices, like the details of their handlooms, their sitting/standing postures while working, etc.

*We thank you for your comments and useful suggestion. We have revised and added of Traditional cloth pulling (Figure 3.2.) A. Traditional cloth pulling*

 (Figure 3.2.)  B. Postures of Praewa silk weavers. *https://f1000research-files.f1000.com/linked/749692.163622-_FRES_%28Figure_3.2.%29%C2%A0_a._Postures_of_Praewa_silk_weavers..png*

 (Figure 3.2.)  B. Postures of Praewa silk weavers. *https://f1000research-files.f1000.com/linked/749693.163622-_FRES_%28Figure_3.2.%29%C2%A0_B._Postures_of_Praewa_silk_weavers..png*

 Methods (Q3): Sharing their dataset and questionnaires will be extremely valuable for other researchers to translate the design improvements to other weaving communities and regions. Suggestion(s):

 > The design process should be documented in more detail, especially the Prototyping stage. How many iterations did the prototype undergo? What changes were made between iterations and how did the researchers decide on what to change?

 > In contrast, the Evaluation aspects of the study are well-detailed, but think the timeline could be clearer since design work often includes some formative evaluation (e.g. between iterations). Did the authors test any of the earlier versions with weavers during prototyping (even informally)?

*We have revised and added in the methodology.*

*Prototyping:1)New models of cloth-pulling devices were developed according to the hand anthropometrics of the weavers and ergonomic design principles to reduce the risk of MSDs during the weaving task. The cloth-pulling devices’ handles were made of plastic polyester materials. Handle length was calculated using the 95 ^th^ percentile of hand length (16.20 cm), handle width using hand circumference (23.10 cm) and handle diameter using grip diameter (4.60 cm). The diameter of a clenched fist was used to set the width of the hand-insertion gap on the handle, which was 4.60 cm. The palm thickness was 1.80 cm, which, when combined for both sides, became 3.60 cm. The hook tip length extending from the handle was 3.38 cm. These measurements were used for 3D modeling of both the front and back views.*

*2)The model was then fabricated as a cloth-pulling device designed according to ergonomic principles.*

*3)The prototype was tested with Praewa silk weavers in two rounds. In the first round, it was found that the handle’s surface was not smooth.*

*4)The researchers thus developed a new prototype with a smooth handle surface, which was then ready for the next stages.*

*In contrast, the Evaluation “ The authors conducted preliminary tests prior to this study; however, these were not included in the research report.*”

 Source Data (Q5): As I noted previously, I really appreciate the data and documentation already included. My suggestions focus on what additional information is needed to replicate the final tool design. Suggestion(s):

 > Include any CAD files that were used in prototype fabrication and list software tools.

 > Bill of materials for the prototype with sources of components/materials.

 Other Suggestions: There are some revisions from previous reviews that have not been addressed.

*We have revised and added in the tool design.*

*Developing new models of pull cloth device according to hand anthropometric of the weavers and ergonomic design principles to reduce the risk of MSDs during the weaving task. The handles were made plastic polyester materials.*

# Reviewer report: Reviewer response for version 2

**DOI:** 10.5256/f1000research.183554.r395094

**Published:** 2025-08-21

**Authors:** Subhasish Sahu

**Affiliations:** 1 Department of Physiology, University of Kalyani Faculty of Science (Ringgold ID: 430677), Kalyani, West Bengal, India

This manuscript discusses a significant issue and offers intriguing research on musculoskeletal stress and ergonomic designing and evaluation of cloth-pulling devices for Praewa Silk Weavers. Overall, the study is well-designed and offers insightful information. However, to increase clarity, technique, and result interpretation, the manuscript might be strengthened in a few places.

 1) Mention the full forms of SD and CV in Table 1.

 2) Explain the selection process of sample size more clearly. If any statistical method was used to determine the selection of sample size, mention it in the methodology section.

 3) Apart from anthropometric measurements, are there any physiological considerations addressed in the design and selection of ergonomic tools? Mention it if it has.

Is the work clearly and accurately presented and does it cite the current literature?

Yes

If applicable, is the statistical analysis and its interpretation appropriate?

Yes

Are all the source data underlying the results available to ensure full reproducibility?

Partly

Is the study design appropriate and is the work technically sound?

Yes

Are the conclusions drawn adequately supported by the results?

Yes

Are sufficient details of methods and analysis provided to allow replication by others?

Partly

Reviewer Expertise:

Ergonomics and Occupational Physiology, Respiratory Physiology, Chronobiology, Oxidative stress, Heat Stress, Organized and unorganized sector

I confirm that I have read this submission and believe that I have an appropriate level of expertise to confirm that it is of an acceptable scientific standard, however I have significant reservations, as outlined above.

# Response

**Published:** 2025-08-28

**Authors:** Teeraphun Kaewdok

**Affiliations:** Faculty of Public Health, Thammasat University, Khlong Luang, Pathum Thani, Thailand

Dear Reviewer, 

 Thank you very much for taking the time to review this manuscript. Please find enclosed our revised manuscript. We thank you the reviewers for their comments. We have revised the manuscript accordingly and provide specific answers.

**Reviewer 3  Version 2**

 Reviewers' comments:

 1) Mention the full forms of SD and CV in Table 1.

 We thank you for your comments and useful suggestion. We have revised and added of SD and CV in Table 1.

**Percentiles:** Indicates the percentage of the population that falls below a certain measurement. For example, the 5 ^th^, 50 ^th ^(mean/median), and 95 ^th^ percentiles are commonly reported.

**Mean (min–max):** The average (mean) value of a measurement, along with its minimum and maximum recorded values in the sample population.

**SD (Standard Deviation):** A measure of the spread or variability in the data.

**CV (Coefficient of Variation):** The standard deviation divided by the mean (usually expressed as a percentage). Indicates the extent of variability in relation to the mean.

 2) Explain the selection process of sample size more clearly. If any statistical method was used to determine the selection of sample size, mention it in the methodology section.

 We have revised and added in the methodology section.

**Sample Size Selection Process.**

 The sample size for this study was determined using statistical power analysis to ensure that the study would have sufficient power to detect a meaningful difference in muscle activity between workstations. The formula described by Heinisch (1965) was applied, which is commonly used to estimate the required sample size when comparing two means. The calculation incorporated the following parameters:

 n = number of samples.

 Zα = The statistical significance level; at the 0.05 level, the value is 1.96.

 Zβ = Corresponds to a test power of 95%, with a value of 1.645.

 ∆ = The difference in mean muscle electrical activity of the weavers between the traditional workstation and the experimental workstation, based on related research. The measurement is expressed in %MVC. The experimental condition involves a seated workstation at a 0-degree seat angle and a 10-degree cloth angle from the horizontal line. The observed difference is 7.97 (Chantaramanee N, Taptagaporn  S,  Piriyaprasarth  P., 2014).

 σ = The standard deviation of the difference in mean  %MVC, with a value of 5.61 (Chantaramanee N, Taptagaporn  S,  Piriyaprasarth  P., 2014).

 The formula used was as follows:

 n= zα+ Zβ (σ)∆ 2 

 Upon substituting the specified values:

 n= *1*. *96*+ *1*. *645* *7*. *97* *5*. *61*2   = (5.12) ^2  ^= 26.22

**

 To prevent data loss and ensure sufficient response from participants, the calculated sample size was increased by 10%,  This result was rounded up, and data were ultimately collected from 29 participants.

 In summary, the sample size was determined using statistical power analysis, specifically the Heinisch (1965) formula for comparing means, with all parameter values based on established standards and previous research findings. This method was chosen to maximize the validity and reliability of the study results.

 3. Apart from anthropometric measurements, are there any physiological considerations addressed in the design and selection of ergonomic tools? Mention it if it has.

 We have revised and added in the physiological considerations in the design and selection of ergonomic tools.

**Physiological measurements.**

 From the study on the working conditions, health impacts, and musculoskeletal risk among Praewa silk weavers, the key findings are as follows:

 1.Working Characteristics and Use of Equipment

 The majority of participants did not use any assistive devices during weaving (77.27%), resulting in repeated manual and arm exertion, which increased their risk of musculoskeletal discomfort and injuries.

 2. Prevalence of Musculoskeletal Disorders

 In the past 12 months, 96.46% of respondents reported musculoskeletal pain or injury related to work, while 84.85% reported such symptoms during the past 7 days. The most commonly affected body parts were the wrists, fingers, and neck.

 3. Postural Risk Assessment

 Assessment of upper limb working posture using 198 observed postures revealed an average Grand Total Score of 6.89±0.31, suggesting a relatively high level of risk for musculoskeletal disorders.

 4. Hand Mobility and Muscle Strength

 The average mobility score of the right hand was 2.90±2.14, which was higher than the left hand (1.99±1.22). Additionally, the grip strength of the dominant hand (0.35±0.07) was greater than that of the non-dominant hand (0.29±0.60), reflecting repetitive use of the dominant limb.

 5. Overall Workplace Safety

 Almost all participants (nearly 99%) perceived a high risk of work-related injuries, which affected their health and quality of life.

 Incorporating physiological data into the design process helps produce tools that are suitable, safe, minimize injuries, and enhance work efficiency. If you need examples of measurement methods or case studies.

# Reviewer report: Reviewer response for version 2

**DOI:** 10.5256/f1000research.183554.r392486

**Published:** 2025-08-19

**Authors:** Ashish Kumar Meher

**Affiliations:** 1 Central University of Jharkhand, Ranchi, Jharkhand, India

After reviewing the revised manuscript, I observed that the authors have not addressed several key points raised in the initial review, particularly in the methodology section. This lack of revision suggests that the feedback may not have been fully considered. Therefore, I do not recommend the indexing of the article in its current form.

Is the work clearly and accurately presented and does it cite the current literature?

Yes

If applicable, is the statistical analysis and its interpretation appropriate?

Yes

Are all the source data underlying the results available to ensure full reproducibility?

Yes

Is the study design appropriate and is the work technically sound?

Yes

Are the conclusions drawn adequately supported by the results?

Yes

Are sufficient details of methods and analysis provided to allow replication by others?

Partly

Reviewer Expertise:

Economics, Development economics, informal sector, handloom industry, occupational health issues

I confirm that I have read this submission and believe that I have an appropriate level of expertise to state that I do not consider it to be of an acceptable scientific standard, for reasons outlined above.

# Reviewer report: Reviewer response for version 1

**DOI:** 10.5256/f1000research.180004.r384478

**Published:** 2025-06-03

**Authors:** Shankar Subramaniam

**Affiliations:** 1 Department of Mechatronics Engineering, Kongu Engineering College | KEC ·, Perundurai, Tamil Nadu, India

- **Abstract Conclusion Improvement**:
  The conclusion section of the abstract should be enhanced by including specific and actionable recommendations based on the study findings.
- **Literature Gap and Study Motivation**:
  Clearly articulate the gap in the existing literature and present the motivation for this study before detailing the methodology section. This will provide a stronger rationale for the research.
- **Novelty of the Work**:
  Given that several similar studies already exist in the textile sector, the manuscript must distinctly highlight the novelty and unique contributions of this research compared to prior works.
- **Participant Age Justification**:
  The rationale behind selecting subjects aged around 70 years needs clarification. Musculoskeletal disorders (MSDs) often manifest with 10–15 years of repetitive work exposure. Hence, including moderately experienced individuals might yield more relevant insights.
- **Description of Work Tasks**:
  The nature of the occupational tasks and work environment should be described first to contextualize the anthropometric and physiological data that follow.
- **Statistical Representation in Charts**:
  Use error bars in all relevant charts to represent variability and statistical significance clearly.
- **Justification for Muscle Selection**:
  Was a questionnaire or preliminary assessment conducted to determine which hand muscles were most affected or relevant? If not, the criteria used to prioritize hand muscles for EMG analysis must be clearly explained.
- **Need for Quantitative Conclusions**:
  The conclusions derived from EMG analysis should be more quantitative and specific, rather than general or observational in nature.

Is the work clearly and accurately presented and does it cite the current literature?

Yes

If applicable, is the statistical analysis and its interpretation appropriate?

Yes

Are all the source data underlying the results available to ensure full reproducibility?

Yes

Is the study design appropriate and is the work technically sound?

Yes

Are the conclusions drawn adequately supported by the results?

Yes

Are sufficient details of methods and analysis provided to allow replication by others?

Yes

Reviewer Expertise:

EMG, Ergonomics

I confirm that I have read this submission and believe that I have an appropriate level of expertise to confirm that it is of an acceptable scientific standard, however I have significant reservations, as outlined above.

# Response

**Published:** 2025-06-10

**Authors:** Teeraphun Kaewdok

**Affiliations:** Faculty of Public Health, Thammasat University, Khlong Luang, Pathum Thani, Thailand

**Dear editor,**

**Thank you very much for taking the time to review this manuscript. Please find enclosed our revised manuscript. We thank you the reviewers for their comments. We have revised the manuscript accordingly and provide specific answers.**

 Abstract Conclusion Improvement:

 The conclusion section of the abstract should be enhanced by including specific and actionable recommendations based on the study findings.

 Response : Thank you for pointing this out. We have revised: The new cloth-pulling device was found to be applicable and acceptable among the weavers. Further research is needed to make appropriate revisions to the ergonomically designed tool based on ergonomic measurements of muscle activity and fatigue under field production conditions. Studies related to workstations and working conditions are needed in the weaving industry sector.

 Literature Gap and Study Motivation:

 Clearly articulate the gap in the existing literature and present the motivation for this study before detailing the methodology section. This will provide a stronger rationale for the research.

 Response : The production of Praewa silk by the Phu Tai community in Ban Phon has experienced significant growth in both quantity and quality. This traditional textile is distinguished by its intricate patterns and substantial dimensions. The price of Praewa silk typically ranges from 15,000 to 40,000 baht per piece. However, certain designs that require a high level of craftsmanship and take approximately 5 to 6 months to complete can command prices between 50,000 and 100,000 baht.

 Novelty of the Work:

 Given that several similar studies already exist in the textile sector, the manuscript must distinctly highlight the novelty and unique contributions of this research compared to prior works.

 Response : The novelty of this research lies in the design of a Praewa silk cloth-pulling devices that adheres to ergonomic principles, specifically tailored for Praewa silk weavers. This innovation aims to enhance worker safety and improve operational efficiency by aligning with the physical capabilities of the users. Furthermore, the device is expected to reduce work-related musculoskeletal discomfort and increase overall production capacity.

 Participant Age Justification:

 The rationale behind selecting subjects aged around 70 years needs clarification. Musculoskeletal disorders (MSDs) often manifest with 10–15 years of repetitive work exposure. Hence, including moderately experienced individuals might yield more relevant insights.

 Response : The inclusion criteria for volunteer participants were set for individuals aged 40 to 70 years. This age range was determined based on the weaving experience commonly found among Praewa  silk weavers. Although many women over the age of 70 still actively engage in Praewa  silk weaving and are capable of sitting and working for extended periods, the research limited the upper age to 70 years to ensure consistency in the study sample. Notably, the average age of participants in this study was 55 years.

 Description of Work Tasks:

 The nature of the occupational tasks and work environment should be described first to contextualize the anthropometric and physiological data that follow.

 Response : The nature of work and working environment in Praewa silk weaving involve repetitive silk-thread pulling in a fixed posture. Weavers typically work 6 to 8 hours per day, performing the same repetitive tasks. This type of work poses a risk of developing wrist-related musculoskeletal disorders, such as carpal tunnel syndrome, De Quervain’s tendonitis, and trigger finger.

 Statistical Representation in Charts:

 Use error bars in all relevant charts to represent variability and statistical significance clearly.

 Justification for Muscle Selection:

 Was a questionnaire or preliminary assessment conducted to determine which hand muscles were most affected or relevant? If not, the criteria used to prioritize hand muscles for EMG analysis must be clearly explained.

 Response : The researcher administered a preliminary questionnaire during Phase I to investigate the use of muscle groups involved in the weaving process. Four muscle groups were examined: the upper trapezius, biceps brachii, brachioradialis, and flexor carpi ulnaris.

# Reviewer report: Reviewer response for version 1

**DOI:** 10.5256/f1000research.180004.r384477

**Published:** 2025-06-03

**Authors:** Ashish Kumar Meher

**Affiliations:** 1 Central University of Jharkhand, Ranchi, Jharkhand, India

Dear editor,

 Thank you for considering me as a potential reviewer for this article. The paper addresses a significant issue concerning MSDs among the handloom weavers. The study is well-designed with respect to its aim and objectives. The findings in this study will be beneficial for improving the musculoskeletal health of the Praewa silk weavers. It will also be helpful for policymakers and future researchers due to its significant contribution to the literature on occupational health and ergonomics. Further, the references are diverse and cover the topic adequately. However, the study has some lacunae, which make it scientifically incomplete. Addressing these issues will enhance the overall quality of this manuscript and make it academically enriched. 

 1. The sample size is very small, and hence, the study findings based on this sample size are a little skeptical in terms of their applicability.

 2. The authors have not mentioned the study region and the sampling framework for drawing the study sample, which is a major drawback of this study.

 3. The rationale for choosing a particular study area also has to be explained.

 4. There is a need for language editing. Many sentences are unclear and incomplete, with grammatical and spelling errors. For example, lines 3-4 of para-1 and the last sentence of para-2 in the introduction section.

 5. **This comment is especially for the editor:** There are many medical terms used in this manuscript. Hence, a recognized medical practitioner or a professor in the field of medicine should also be chosen as a second reviewer to look into the validity and applicability of these terms and their significance.

Is the work clearly and accurately presented and does it cite the current literature?

Yes

If applicable, is the statistical analysis and its interpretation appropriate?

Yes

Are all the source data underlying the results available to ensure full reproducibility?

Yes

Is the study design appropriate and is the work technically sound?

Yes

Are the conclusions drawn adequately supported by the results?

Yes

Are sufficient details of methods and analysis provided to allow replication by others?

Partly

Reviewer Expertise:

Economics, Development economics, informal sector, handloom industry, occupational health issues

I confirm that I have read this submission and believe that I have an appropriate level of expertise to confirm that it is of an acceptable scientific standard, however I have significant reservations, as outlined above.

# Response

**Published:** 2025-06-10

**Authors:** Teeraphun Kaewdok

**Affiliations:** Faculty of Public Health, Thammasat University, Khlong Luang, Pathum Thani, Thailand

**Dear editor,**

**Thank you very much for taking the time to review this manuscript. Please find enclosed our revised manuscript. We thank you the reviewers for their comments. We have revised the manuscript accordingly and provide specific answers.**

 1. The sample size is very small, and hence, the study findings based on this sample size are a little skeptical in terms of their applicability.

 Response : Thank you for pointing this out. The population in this study consists of individuals engaged in the production of Praewa silk weavers. The sample size was calculated using the formula by Heinisch (1965).

 n= zα+ Zβ (σ)∆ 2 

 n= *1*. *96*+ *1*. *645* *7*. *97* *5*. *61*2 

 n= *28*. *731* *5*. *61* 2 

 n= *26*.22 ******

**

 n = number of samples.

 Zα = The statistical significance level; at the 0.05 level, the value is 1.96.

 Zβ = Corresponds to a test power of 95%, with a value of 1.645.

 ∆ = The difference in mean muscle electrical activity of the weavers between the traditional workstation and the experimental workstation, based on related research. The measurement is expressed in %MVC. The experimental condition involves a seated workstation at a 0-degree seat angle and a 10-degree cloth angle from the horizontal line. The observed difference is 7.97 (Chantaramanee N, Taptagaporn  S,  Piriyaprasarth  P., 2014).

 σ = The standard deviation of the difference in mean  %MVC, with a value of 5.61 (Chantaramanee N, Taptagaporn  S,  Piriyaprasarth  P., 2014).

 To prevent data loss and ensure sufficient response from participants, the calculated sample size was increased by 10%, resulting in a total of 28.6. Therefore, the study collected data from a total of 29 participants.

 2. The authors have not mentioned the study region and the sampling framework for drawing the study sample, which is a major drawback of this study.

 Response : Thank you for pointing this out. This quasi-experimental research was conducted as Phase III of the study. In Phase I, the population consisted of participants from four provinces in Thailand: Kalasin, Sakon Nakhon, Nakhon Phanom, and Mukdahan. In Phase III, participants were selected using purposive sampling, focusing on the province with the highest number of Praewa silk weavers, which was Kalasin Province. A total of 29 volunteers were assigned to three experimental conditions using the block randomization method: (A) traditional cloth pulling, (B) using a standard cloth-pulling device and (C) using an ergonomic prototype cloth-pulling device.

 3. The rationale for choosing a particular study area also has to be explained.

 Response : Since the of Praewa silk weavers requires consistent patterns and is conducted under the same experimental conditions within the same area, it was necessary to select a single location for the experiment. Each volunteer participated in the experimental procedures at the site for approximately 295 minutes.

 4. There is a need for language editing. Many sentences are unclear and incomplete, with grammatical and spelling errors. For example, lines 3-4 of para-1 and the last sentence of para-2 in the introduction section.

 Response : Language editing in lines 3-4 of para-1(There were 18.47 million people (31.35%) outside the labor force in Thailand, of whom skilled workers accounted for 10.68%.)

 Language editing in last sentence of para-2 in the introduction section. (The study aims to design an ergonomics-based cloth-pulling device and to evaluate the effectiveness of such devices for Praewa silk weavers.)
